# Erythrocyte-derived microvesicles induce arterial spasms in *JAK2^V617F^* myeloproliferative neoplasm

**DOI:** 10.1172/JCI124566

**Published:** 2020-04-20

**Authors:** Johanne Poisson, Marion Tanguy, Hortense Davy, Fatoumata Camara, Marie-Belle El Mdawar, Marouane Kheloufi, Tracy Dagher, Cécile Devue, Juliette Lasselin, Aurélie Plessier, Salma Merchant, Olivier Blanc-Brude, Michèle Souyri, Nathalie Mougenot, Florent Dingli, Damarys Loew, Stephane N. Hatem, Chloé James, Jean-Luc Villeval, Chantal M. Boulanger, Pierre-Emmanuel Rautou

**Affiliations:** 1 Paris–Centre de recherche cardiovasculaire (PARCC), Université de Paris, Paris, France.; 2 Centre de recherche sur l’inflammation, Inserm, Université de Paris, Paris, France.; 3 Geriatrics Department, Hôpital Européen Georges Pompidou, Assistance Publique–Hôpitaux de Paris (AP-HP), Paris, France.; 4 Inserm U1170, Institut Gustave Roussy, Université Paris XI, Villejuif, France.; 5 Service d’Hépatologie, Pôle des Maladies de l’Appareil Digestif, Hôpital Beaujon, Département Hospitalo-Universitaire (DHU Unity), AP-HP, Clichy, France.; 6 Centre de Référence des Maladies Vasculaires du Foie, French Network for Rare Liver Diseases (FILFOIE), European Reference Network (ERN), Clichy, France.; 7 Inserm UMR S1131, University Hospital Institute (IHU), Université de Paris, Paris, France.; 8 Inserm UMS 28, Phénotypage du petit animal, Plateforme d’expérimentations coeur-muscle-vaisseaux (PECMV), Sorbonne University, Paris, France.; 9 Laboratoire de Spectrométrie de Masse Protéomique, Institut Curie, Université de recherche PSL, Paris, France.; 10 Inserm, UMR 1166, Institut de cardiométabolisme et nutrition (ICAN), Sorbonne University, Paris, France.; 11 Inserm U1034, Biology of Cardiovascular, Pessac, France.; 12 University of Bordeaux, Pessac, France.; 13 Laboratory of Hematology, Bordeaux University Hospital Center, Pessac, France.

**Keywords:** Cardiology, Hematology, Cancer, Cardiovascular disease, endothelial cells

## Abstract

Arterial cardiovascular events are the leading cause of death in patients with *JAK2^V617F^* myeloproliferative neoplasms (MPNs). However, their mechanisms are poorly understood. The high prevalence of myocardial infarction without significant coronary stenosis or atherosclerosis in patients with MPNs suggests that vascular function is altered. The consequences of *JAK2^V617F^* mutation on vascular reactivity are unknown. We observe here increased responses to vasoconstrictors in arteries from *Jak2^V617F^* mice resulting from a disturbed endothelial NO pathway and increased endothelial oxidative stress. This response was reproduced in WT mice by circulating microvesicles isolated from patients carrying *JAK2^V617F^* and by erythrocyte-derived microvesicles from transgenic mice. Microvesicles of other cellular origins had no effect. This effect was observed ex vivo on isolated aortas, but also in vivo on femoral arteries. Proteomic analysis of microvesicles derived from *JAK2^V617F^* erythrocytes identified increased expression of myeloperoxidase as the likely mechanism accounting for their effect. Myeloperoxidase inhibition in microvesicles derived from *JAK2^V617F^* erythrocytes suppressed their effect on oxidative stress. Antioxidants such as simvastatin and *N*-acetyl cysteine improved arterial dysfunction in *Jak2^V617F^* mice. In conclusion, *JAK2^V617F^* MPNs are characterized by exacerbated vasoconstrictor responses resulting from increased endothelial oxidative stress caused by circulating erythrocyte-derived microvesicles. Simvastatin appears to be a promising therapeutic strategy in this setting.

## Graphical abstract

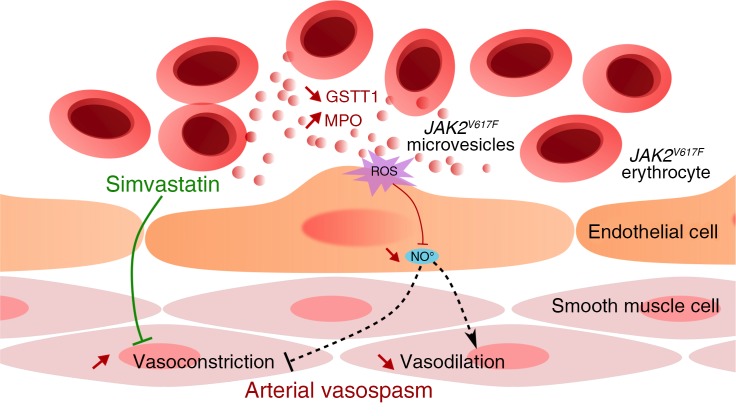

## Introduction

Bcr/Abl-negative myeloproliferative neoplasms (MPNs) are clonal hematopoietic stem cell disorders characterized by the proliferation of particular hematopoietic lineages without blockage in cell maturation. They include polycythemia vera, essential thrombocythemia, and primary myelofibrosis (1). *JAK2* is the most common MPN driver gene. *JAK2^V617F^* is a gain-of-function mutation that leads to growth factor hypersensitivity, detected in about 70% of MPNs (95% in polycythemia vera and 50%–60% in essential thrombocythemia and prefibrotic/primary myelofibrosis) (1). *JAK2^V617F^* appears in pluripotent hematopoietic progenitor cells and is present in all erythroid and myeloid lineages (1). In addition, several groups have described *JAK2^V617F^* in endothelial cells in the liver and spleen of patients with splanchnic vein thrombosis (2, 3) and in circulating endothelial progenitor cells (4–6).

About 30% of MPNs are revealed by cardiovascular events. Cardiovascular diseases (CVDs) are the first cause of morbidity and mortality in patients with MPNs (7). Arterial events represent 60%–70% of these cardiovascular events (7). Interestingly, myocardial infarction without significant coronary stenosis by angiography was observed in 21% of patients with MPN (8) versus 3% in a similar population without MPN (9). This observation prompted the European Society of Cardiology to recommend searching for MPNs in the case of myocardial infarction without obstructive coronary artery disease (10). The mechanism underlying this link between myocardial infarction without obstructive coronary artery disease and MPNs is unknown, but a vasoactive phenomenon (local intense vasoconstriction) is suspected (11, 12). Therefore, the purpose of the present study was to examine the consequences of *JAK2^V617F^* mutation on arterial vascular reactivity.

## Results

### Increased arterial contraction in mice carrying Jak2^V617F^ in hematopoietic and endothelial cells.

**Figure 1:**
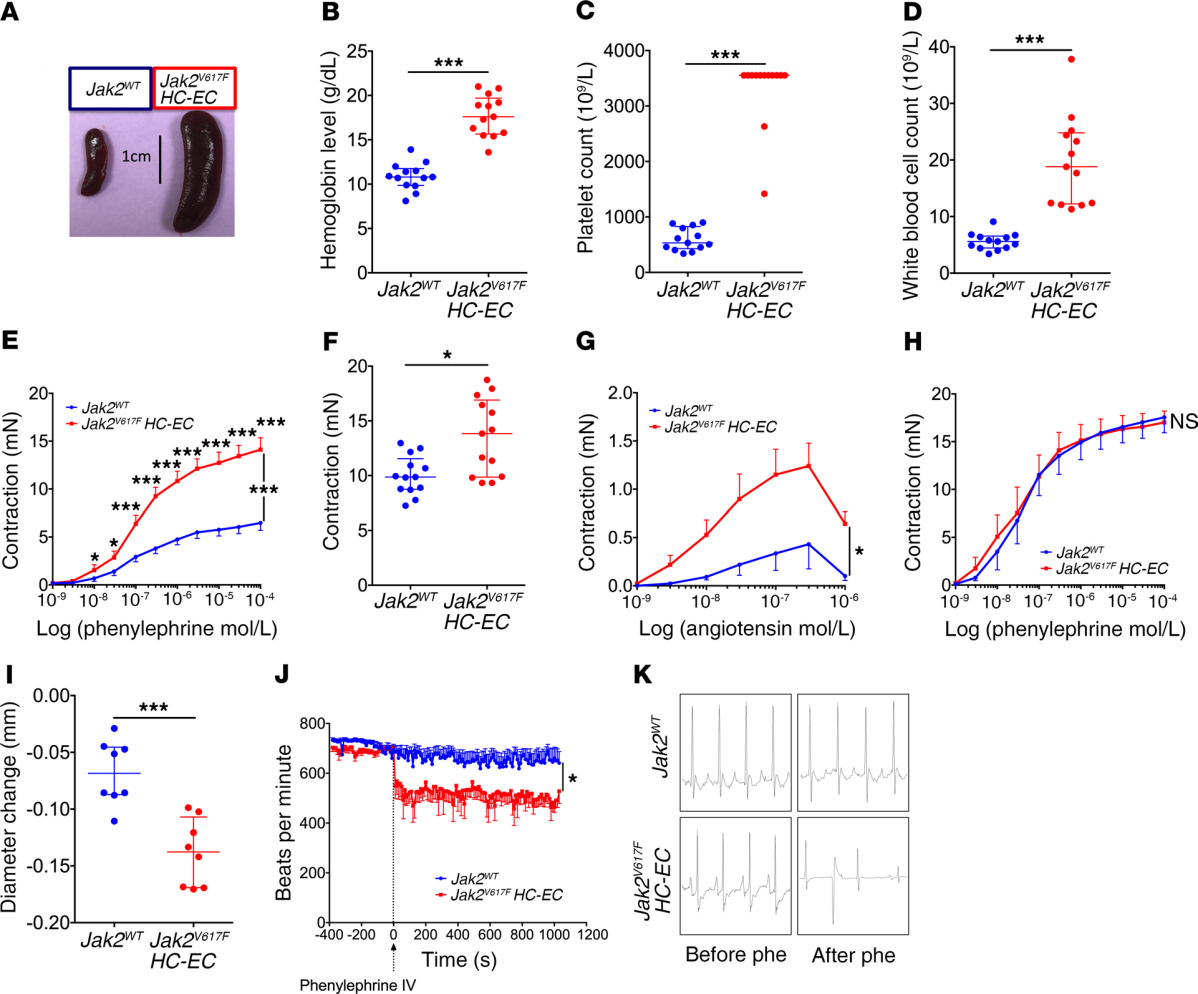
JAK2^V617F^ in hematopoietic and endothelial cells increases arterial contraction in an endothelium-dependent manner. (**A**) Representative image of the spleen. Hemoglobin level (**B**), platelet count (**C**), and WBC count (**D**) of 8 -to 12-week-old control mice (*Jak2^WT^*, *n* = 13) and *Jak2^V617F Flex/WT^ VE-cadherin-Cre* mice (*Jak2^V617F^ HC-EC*, *n* = 13). Cumulative dose-response curves to phenylephrine (*Jak2^WT^*, *n* = 13; *Jak2^V617F^ HC-EC*, *n* = 13) (**E**) and to angiotensin II (*Jak2^WT^*, *n* = 3; *Jak2^V617F^ HC-EC*, *n* = 4) (**G**), and contraction response to potassium chloride (80 mmol/L) (*Jak2^WT^*, *n* = 13; *Jak2^V617F^ HC-EC*, *n* = 13) of aortas with endothelium (**F**). (**H**) Cumulative dose-response curves to phenylephrine of aortas without endothelium (*Jak2^WT^*, *n* = 6; *Jak2^V617F^ HC-EC*, *n* = 6). (**I**) Diameter change of femoral artery after phenylephrine injection (10^–3^ mol/L) (*Jak2^WT^*, *n* = 8; *Jak2^V617F^ HC-EC*, *n* = 8). Electrocardiogram recording before and after intravenous phenylephrine (Phe) injection (3 mg/kg; *Jak2^WT^*, *n* = 13; *Jak2^V617F^ HC-EC*, *n* = 6) (**J**), with representative images of the changes observed in 5 of 6 *Jak2^V617F^ HC-EC* versus 4 of 13 *Jak2^WT^* mice (*P* = 0.057) (**K**). Quantitative data are expressed as median with IQR, and cumulative dose-response curves are expressed as mean with SEM. *P < 0.05, ***P < 0.001. Cumulative dose-response curves and electrocardiogram recordings were compared using ANOVA for repeated measures, and other data were compared using the Mann-Whitney *U* test. All tests were 2 sided.

As *JAK2^V617F^* is present in both hematopoietic and endothelial cells in patients with MPN (2, 3), we first investigated vasoactive response in a mouse model mimicking the human disease. We generated mice expressing JAK2^V617F^ in hematopoietic and in endothelial cells by crossing *Jak2^V617F Flex/WT^* mice with *VE-cadherin-Cre* mice. As expected, *Jak2^V617F Flex/WT^ VE-cadherin-Cre* mice, herein referred to as *Jak2^V617F^ HC-EC* mice, in which *VE-cadherin* was expressed during early embryonic life in a precursor of both endothelial and hematopoietic cells (13), developed MPN, as attested by higher spleen weight (2.3%–5.7% of body weight vs. 0.3%–0.6% for littermate controls; *P* < 0.0001) and higher hemoglobin levels and platelet and WBC counts than in littermate controls (Figure 1, A–D). Endothelial and hematopoietic progenitor cell recombination was verified by crossing *VE-cadherin-Cre* with *mTmG* mice (Supplemental Figure 1; supplemental material available online with this article; https://doi.org/10.1172/JCI124566DS1).

In myography assays, we observed ex vivo that aortas from *Jak2^V617F^ HC-EC* mice displayed a substantial increase in the response not only to phenylephrine (Figure 1E), but also to potassium chloride (Figure 1F) and angiotensin II (Figure 1G), as compared with littermate controls. Removing the endothelium suppressed this increased arterial contraction (Figure 1H). Likewise, we observed in vivo an increased response to phenylephrine in femoral arteries from *Jak2^V617F^ HC-EC* mice compared with littermate controls (Figure 1I). Thus, *Jak2^V617F^* in endothelial and hematopoietic cells strongly increases arterial response to vasoconstrictors in an endothelium-dependent manner.

Because of the high rate of myocardial infarction without significant coronary stenosis reported in patients with MPN (8), we investigated the cardiac vascular bed by performing electrocardiography in *Jak2^V617F^ HC-EC* mice and their littermate controls. After intravenous injection of phenylephrine, *Jak2^V617F^ HC-EC* mice showed electrocardiogram modifications, including bradycardia and arrhythmia, that are indirect signs of coronary spasm (ref. 14 and Figure 1, J and K).

### Increased arterial contraction in mice with JAK2^V617F^ specifically expressed in hematopoietic cells but not in endothelial cells.

**Figure 2:**
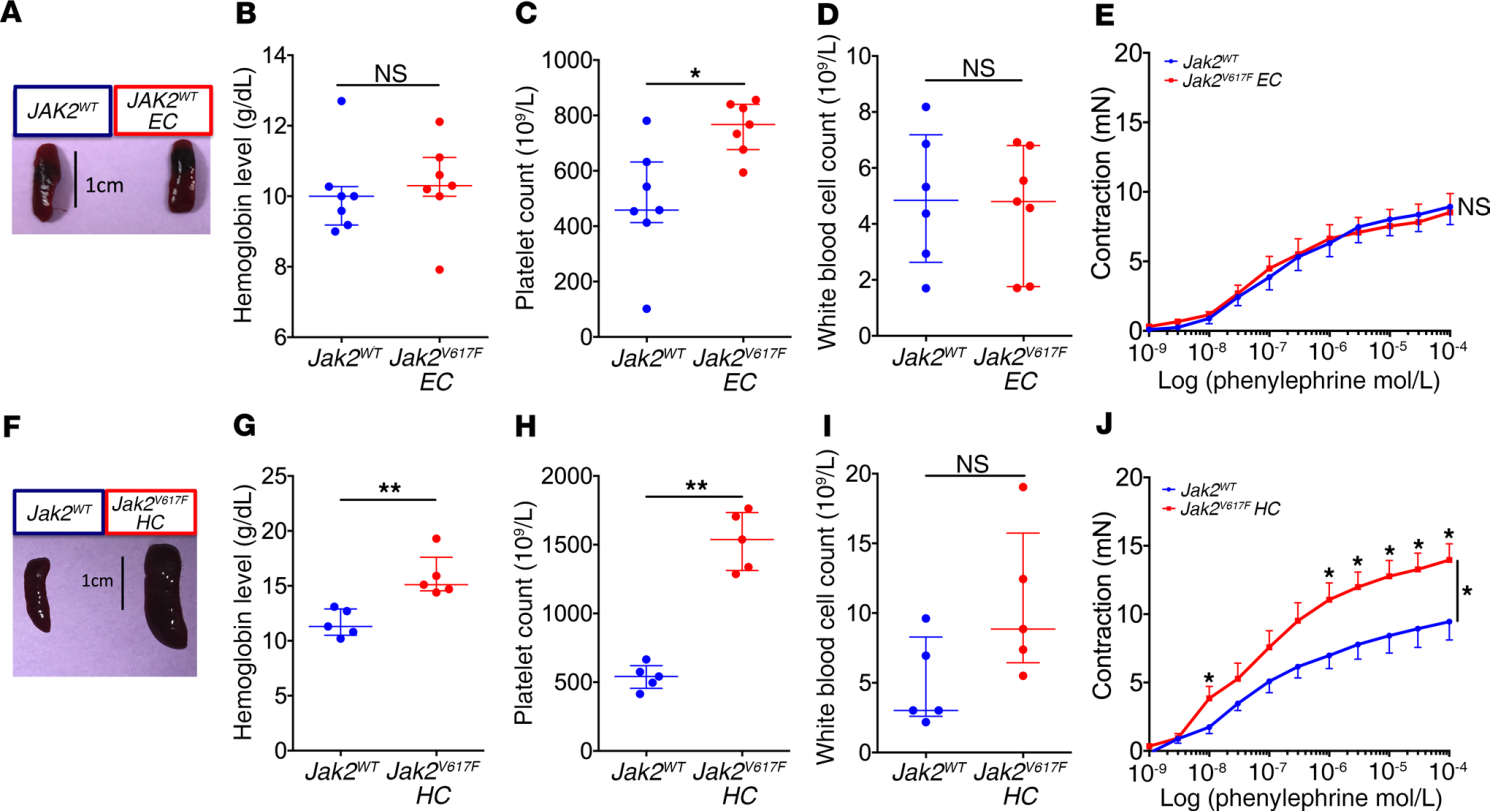
JAK2^V617F^ specifically expressed in hematopoietic, but not endothelial, cells increases arterial contraction. (**A** and **F**) Representative images of the spleen. Blood cell count of 10- to 13-week-old control mice (*Jak2^WT^*, *n* = 7) and *Jak2^V617F Flex/WT^ VE-cadherin-Cre-ERT2* mice (*Jak2^V617F^ EC*, *n* = 7) (**B**–**D**) and of 13- to 15-week-old chimeric C57BL/6 mice transplanted with bone marrow of WT mice (*Jak2^WT^*, *n* = 5) or of *Jak2^V617F^ HC-EC* mice (*Jak2^V617F^ HC*, *n* = 5) (**G**–**I**). Data are expressed as median with IQR. Cumulative dose-response curve to phenylephrine of aortas from *Jak2^WT^* (*n* = 7) and *Jak2^V617F^ EC* mice (*n* = 7) (**E**) and from *Jak2^WT^* (*n* = 5) and *Jak2^V617F^ HC* mice (*n* = 5) (**J**). Quantitative data are expressed as median with IQR, and cumulative dose-response curves are expressed as mean with SEM. *P < 0.05, **P < 0.01. Cumulative dose-response curves were compared using ANOVA for repeated measures, and other data were compared using the Mann-Whitney *U* test. All tests were 2 sided.

To determine whether this increased arterial contraction was due to JAK2^V617F^ in endothelial cells or in hematopoietic cells, we first generated mice expressing JAK2^V617F^ only in endothelial cells. We crossed *Jak2^V617F Flex/WT^* mice with inducible *VE-cadherin-Cre-ERT2* mice expressing the Cre recombinase after tamoxifen injection only in endothelial cells. As expected, *Jak2^V617F Flex/WT^* VE-cadherin-Cre-ERT2 (herein referred to as *Jak2^V617F^ EC*) mice did not develop MPN (Figure 2, A–D). Adequate endothelial recombination was verified by crossing *VE-cadherin-Cre-ERT2* mice with *mTmG* mice (Supplemental Figure 1). We previously demonstrated the absence of hematopoietic recombination in this model (15). We observed no difference in arterial response to phenylephrine between the *Jak2^V617F^ EC* mice and littermate controls (*Jak2^WT^*) (Figure 2E).

To assess the implication of JAK2^V617F^ in hematopoietic cells, we generated mice expressing *Jak2^V617F^* only in hematopoietic cells by transplanting into lethally irradiated C57BL/6 mice *Jak2^V617F^* bone marrow cells obtained from *Jak2^V617F^ HC-EC* mice. Irradiated C57BL/6 mice transplanted with *Jak2^WT^* BM cells were used as controls. Hematopoietic expression of JAK2^V617F^ induced an MPN (Figure 2, F–I) and an increased arterial response to phenylephrine (Figure 2J). Taken together, these findings demonstrate that the presence of the *Jak2^V617F^* mutation in hematopoietic, but not endothelial, cells is responsible for the increase in arterial contraction in response to vasoconstrictors we observed in *Jak2^V617F^ HC-EC* mice (Figure 1, E–G).

### Increased arterial contraction induced by microvesicles from JAK2^V617F^ patients.

**Figure 3:**
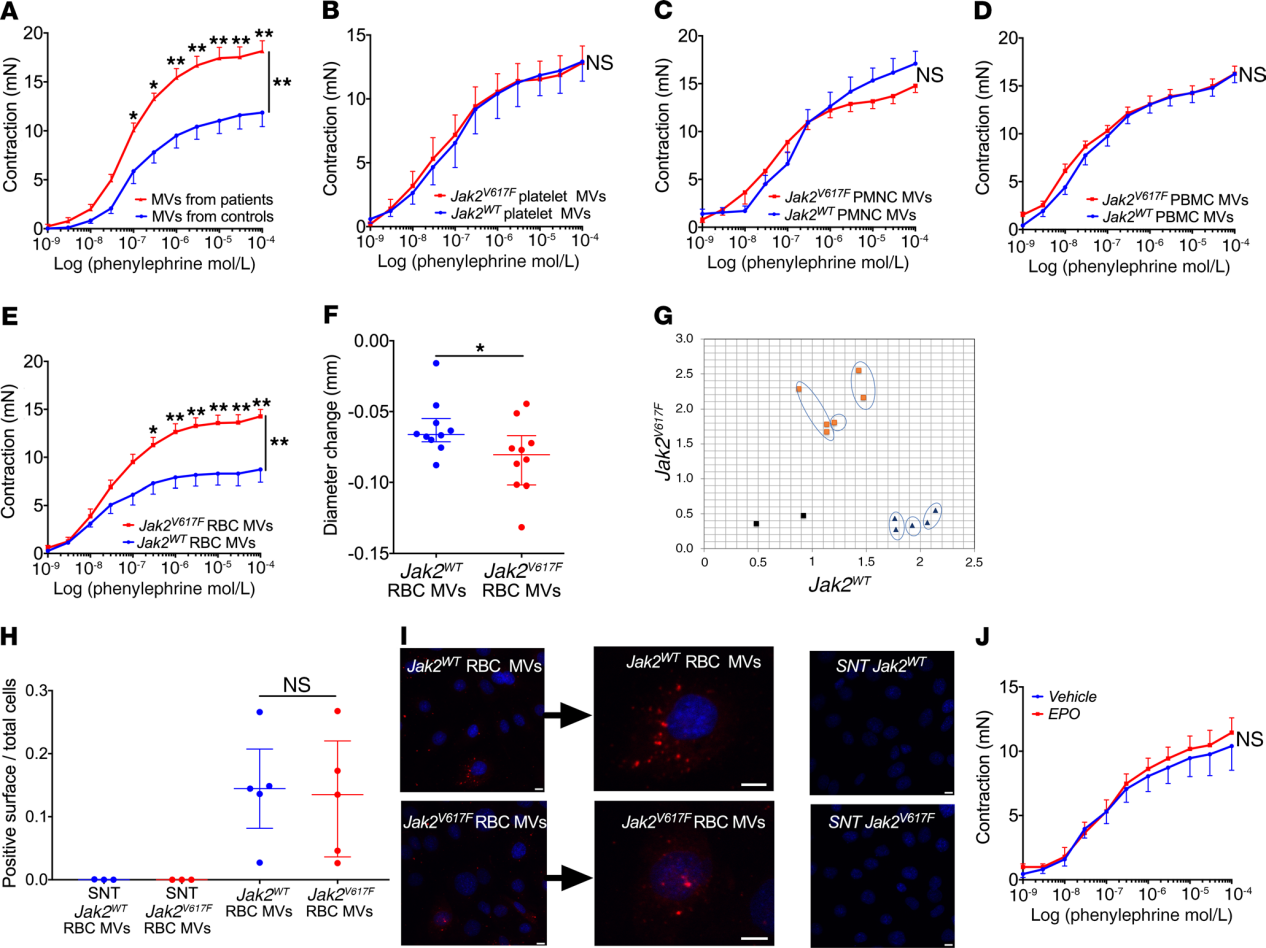
Microvesicles derived from JAK2^V617F^ RBCs are responsible for increased arterial contraction. (**A**) Cumulative dose-response curves to phenylephrine of aortas from WT mice incubated with microvesicles (MVs) isolated from JAK2^V617F^ patients (*n* = 7) and control individuals (*n* = 5) at their circulating concentration. Cumulative dose-response curves to phenylephrine of aortas from WT mice incubated with microvesicles generated from platelets (*n* = 5 and *n* = 5, respectively) (**B**), PBMCs (*n* = 5 and *n* = 6, respectively) (**C**), PMNCs (*n* = 5 and *n* = 5) (**D**), and RBCs (*n* = 9 and *n* = 4, respectively) (**E**) from *Jak2^V617F^ HC-EC* mice (*Jak2^V617F^*) or littermate control mice (WT). (**F**) Change in diameter of femoral artery induced by phenylephrine injection (10^–3^ mol/L) in control mice previously injected with control erythrocyte–derived microvesicles (*Jak2^WT^* RBC MVs, *n* = 10) or with *Jak2^V617F^* erythrocyte–derived microvesicles (*Jak2^V617F^* RBC MVs , *n* = 10). (**G**) Allelic discrimination plot of ARN isolated from microvesicles derived from *Jak2^WT^* (in blue) and *Jak2^V617F^* erythrocytes (in orange) (*n* = 3 per group); no template control (NTC), black. Quantification (**H**) and representative images (**I**) of the uptake by endothelial cells (HUVECs with DAPI in blue) of erythrocyte-derived microvesicles from *JAK2^V617F^* mice (*n* = 5) or *JAK2^WT^* mice (*n* = 5) or respective 20,500-*g* supernatant (SNT; *n* = 3 for each group). Scale bars: 10 μm. (**J**) Cumulative dose-response curve to phenylephrine of aortas from WT mice injected with vehicle (*n* = 5) or with epoetin (EPO; *n* = 8). Quantitative data are expressed as median with IQR, and cumulative dose-response curves are expressed as mean with SEM. *P < 0.05, **P < 0.01. Cumulative dose-response curves were compared using ANOVA for repeated measures, and other data were compared using the Mann-Whitney *U* test. All tests were 2 sided.

We then sought to identify the mediators responsible for the increased response to vasoconstrictors when JAK2^V617F^ was present in hematopoietic cells and tested the hypothesis that circulating blood might convey biological information from hematopoietic cells to the vascular wall. Circulating microvesicles, i.e., extracellular vesicles having a size ranging from 0.1 to 1 μm, are now recognized as triggers of various types of vascular dysfunction (16). We therefore examined the effect of circulating microvesicles isolated from the blood of patients with MPN on vascular responses to vasoactive agents. We isolated plasma microvesicles from 7 patients carrying JAK2^V617F^ (2 males, 5 females; blood drawn before introduction of cytoreductive therapy) and from 5 healthy control individuals (2 males, 3 females; age not significantly different from that of patients). We incubated these microvesicles at their plasma concentration with aortic rings from WT mice and observed that plasma microvesicles from patients carrying JAK2^V617F^ reproduced the increased response to phenylephrine (Figure 3A). Plasma without microvesicles from the same patients and controls had no effect (data not shown).

### Increased arterial contraction induced by erythrocyte-derived microvesicles from Jak2^V617F^ mice.

We then sought to determine the subpopulation of microvesicles responsible for this increased arterial contraction. We generated microvesicles from each type of blood cell from *Jak2^V617F^ HC-EC* mice or littermate controls and incubated these microvesicles, at the same concentration, with aortic rings from WT mice. Erythrocyte-derived microvesicles generated from *Jak2^V617F^ HC-EC* mice reproduced the increased response to phenylephrine on aortic rings ex vivo (Figure 3E), while platelet, PBMC, and polymorphonuclear cell (PMNC) microvesicles did not (Figure 3, B–D). Likewise, in vivo, femoral arteries from WT mice injected with microvesicles derived from JAK2^V617F^ erythrocytes displayed an increased response to phenylephrine as compared with WT mice injected with microvesicles derived from erythrocytes of littermate controls (Figure 3F). Microvesicles generated from *Jak2^V617F^ HC-EC* mouse erythrocytes carried *Jak2^V617F^* mRNA (Figure 3G). To investigate the interaction of erythrocyte-derived microvesicles with endothelial cells, we labeled microvesicles with the fluorescent dye PKH26, incubated them with endothelial cells, and performed confocal microscopy on endothelial cells. Fluorescence was detected in endothelial cells, suggesting that erythrocyte-derived microvesicles were taken up by endothelial cells (Figure 3, H and I, and ref. 17). No difference in uptake was observed between microvesicles from *Jak2^V617F^ HC-EC* erythrocytes and their WT counterparts (Figure 3, H and I).

We then sought to determine whether the increased number of erythrocytes could in itself explain this effect or if qualitative changes were involved. We generated a mouse model of polycythemia without *Jak2^V617F^* caused by chronic epoetin injections. After 3 weeks of epoetin treatment, hemoglobin reached a level similar to that in *Jak2^V617F^ HC-EC* mice (18.5 g/dL, IQR 16.5–19.5, vs. 17.6 g/dL, IQR 15.7–19.7; *n* = 5 and *n* = 13, respectively; *P* = 0.67). However, this model with a high number of circulating erythrocytes failed to reproduce the increased response to phenylephrine observed in *Jak2^V617F^ HC-EC* mouse aortas (Figure 3J). Thus, the presence of the *Jak2^V617F^* mutation in erythrocyte-derived microvesicles was required to cause increased arterial contraction.

### NO pathway inhibition and increased endothelial oxidative stress.

**Figure 4:**
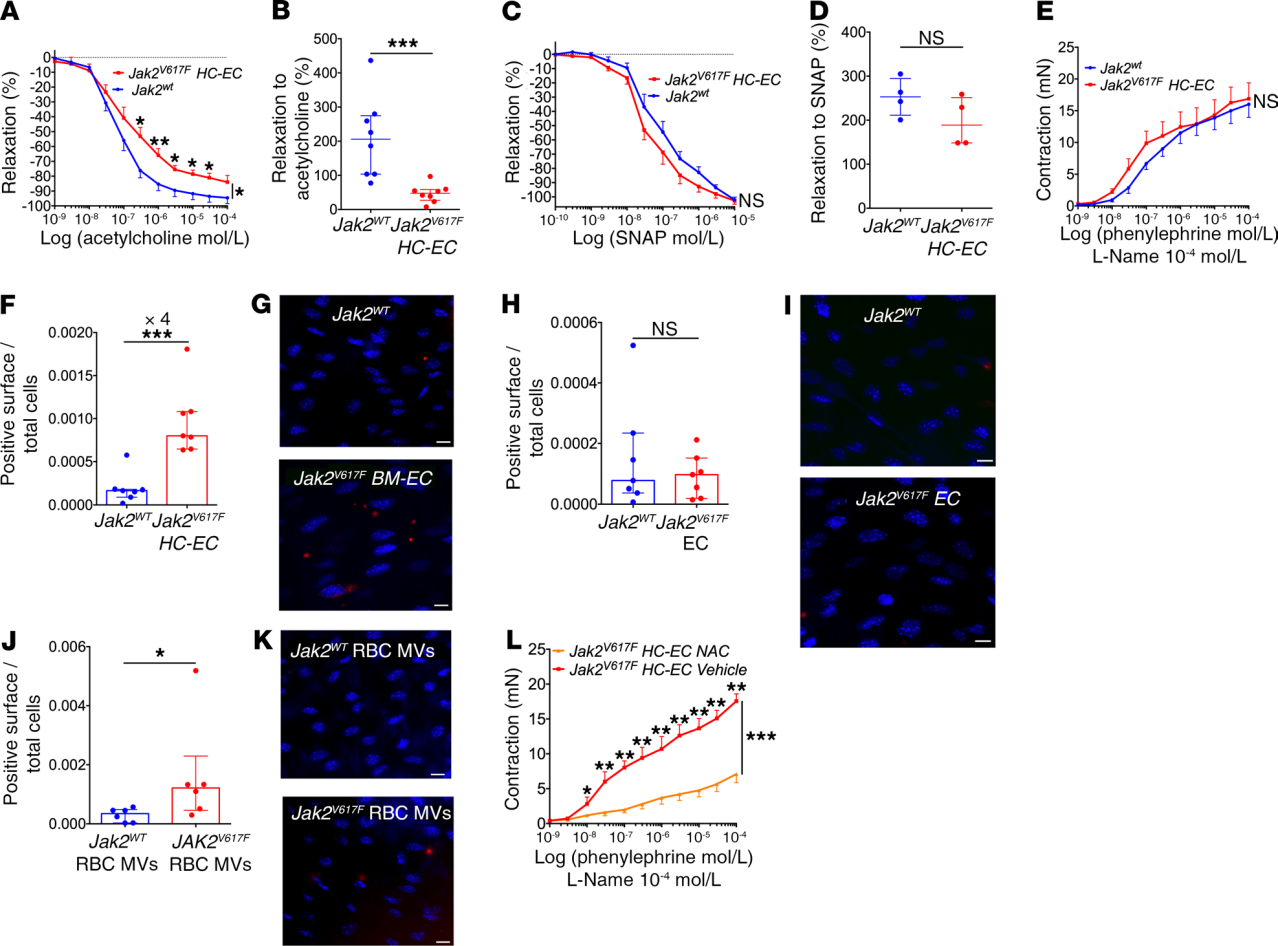
Disturbed endothelial NO pathway and increased oxidative stress status. Cumulative dose-response curve of aortas from *Jak2^V617F^ HC-EC* mice and littermate controls (*Jak2^WT^*) to acetylcholine (*n* = 11 and *n* = 11, respectively) (**A**) and to SNAP (*n* = 5 and *n* = 6, respectively) (**C**), and to phenylephrine after l-NAME incubation (*n* = 11 and *n* = 7, respectively) (**E**). Diameter change of femoral arteries after injection of acetylcholine (10^–2^ mol/L) (*Jak2^WT^*, *n* = 8; *Jak2^V617F^ HC-EC,* *n* = 8) (**B**) and SNAP (10^–3^ mol/L) (*Jak2^WT^*, *n* = 4; *Jak2^V617F^ HC-EC*, *n* = 4) (**D**). Quantification of ROS generation (red surface) per endothelial cell in control mice (*Jak2^WT^*) versus *Jak2^V617F^ HC-EC* mice (**F**); control mice (*Jak2^WT^*) versus *Jak2^V617F^ EC* mice (**H**); control mice injected with microvesicles derived from control (*Jak2^WT^* RBC MVs, *n* = 6) or *JAK2^V617F^* erythrocytes (*Jak2^V617F^* RBC MVs, *n* = 6) (**J**). Representative images of en face endothelial staining with CellROX (red fluorogenic probes for ROS generation) and DAPI (nuclei in blue) of aortas (**G**, **I**, and **K**). Scale bars: 10 μm. (**L**) Cumulative dose-response curve to phenylephrine of aortas from *Jak2^V617F^ HC-EC* mice treated with vehicle (*n* = 5) and with NAC (*n* = 7). *P < 0.05, **P < 0.01, ***P < 0.001. Quantitative data are expressed as median with IQR and compared using the Mann-Whitney *U* test, and cumulative dose-response curves are expressed as mean with SEM and compared using ANOVA for repeated measures.

We then investigated how microvesicles derived from JAK2^V617F^ erythrocytes increase the response to vasoconstrictive agents. We first examined the NO pathway. We observed ex vivo on aortas and in vivo on femoral arteries that dilatation was impaired in response to acetylcholine in *Jak2^V617F^ HC-EC* mice, reproducing the human disease (Figure 4, A and B). This impaired dilatation capacity was not due to decreased sensitivity to NO of vascular smooth muscle cells, as the response to a direct NO donor (*S*-nitroso-*N*-acetyl-dl-penicillamine [SNAP]) was not different between *Jak2^V617F^ HC-EC* mice and littermate controls, either ex vivo or in vivo (Figure 4, C and D). We also observed that, after preincubation with the NOS inhibitor l-NAME, aortas from *Jak2^V617F^ HC-EC* mice had a response to phenylephrine similar to that of littermate controls (Figure 4E). Therefore, these results demonstrate that the increased arterial contraction observed in *Jak2^V617F^ HC-EC* mice resulted from a dysfunctional endothelial NO pathway.

Because previous work showed that heme in erythrocyte microvesicles can scavenge NO (18, 19), we quantified heme in microvesicles derived from *Jak2^V617F^ HC-EC* mice and control mouse erythrocytes, but observed no difference (Supplemental Figure 2).

We then investigated generation of ROS, i.e., inhibitors of NOS activity and NO bioavailability (20), and found that there was 4 times more ROS in the aortic endothelium of *Jak2^V617F^ HC-EC* mice than that of littermate controls (Figure 4, F and G). Conversely, ROS generation was normal in the aortic endothelium of *Jak2^V617F^* EC mice, expressing JAK2^V617F^ only in endothelial cells (Figure 4, H and I). Likewise, aortic endothelium from WT mice injected with microvesicles derived from JAK2^V617F^ erythrocytes generated more reactive species than aortic endothelium from mice injected with microvesicles derived from littermate control erythrocytes (Figure 4, J and K). There was no ROS generation in underlying smooth muscle cells in any of these experiments (data not shown). Together, these results show that microvesicles derived from JAK2^V617F^ erythrocytes induce excessive oxidative stress in endothelial cells, leading to decreased availability of NO.

To ascertain the implication of the increased oxidative stress in the increased arterial contraction in *Jak2^V617F^ HC-EC* mice, we treated these mice with NAC (activator of the glutathione pathway and an antioxidant) for 14 days intraperitoneally. This treatment had no effect on blood cell count or spleen weight (Supplemental Figure 4) but normalized arterial contraction in response to phenylephrine (Figure 4L).

### Increased endothelial oxidative stress status by myeloperoxidase in erythrocyte-derived microvesicles from Jak2^V617F^ mice.

**Figure 5:**
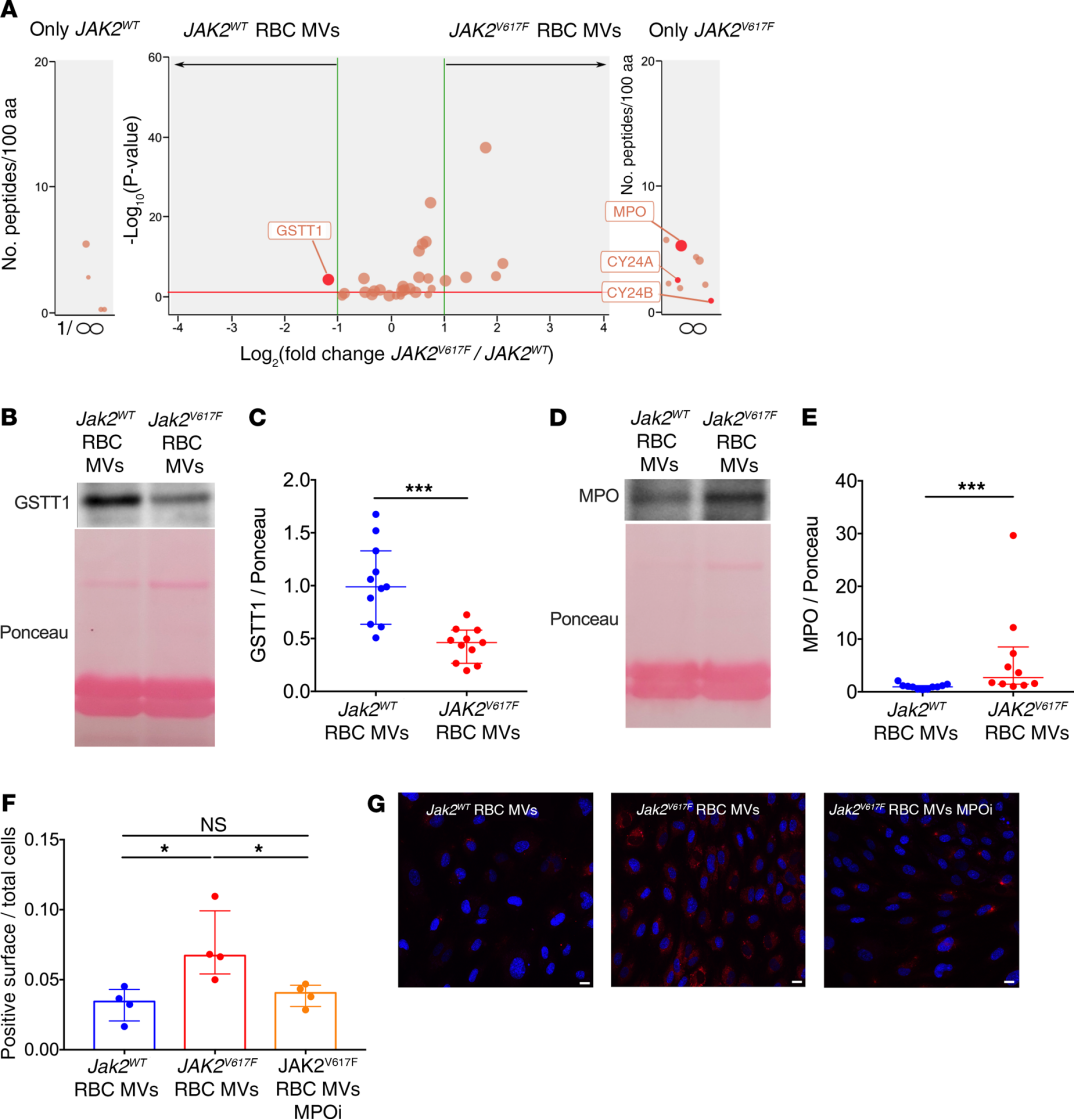
MPO carried by erythrocyte-derived microvesicles from *Jak2^V617F^* mice is responsible for increased endothelial oxidative stress. (**A**) Volcano plot obtained by using quantitative label-free mass spectrometry analysis of proteins isolated from microvesicles derived from *JAK2^V617F^* (*n* = 6) and *JAK2^WT^* (*n* = 4) erythrocytes (ratio *JAK2^V617F^*/*JAK2^WT^*); only proteins involved in cellular oxidant detoxification (GO 0098869) and ROS metabolic process (GO 0072593) are presented (red line corresponds to *P* = 0.05). Representative image of GSTT1 (**B**) and MPO (**D**) Western blots performed on erythrocyte-derived microvesicles, with respective quantification (**C** and **E**) (*JAK2^WT^* erythrocyte microvesicles, *n* = 11; *JAK2^V617F^* erythrocyte microvesicles, *n* = 11). (**F**) Quantification of ROS generation (red surface) per endothelial cell (HUVEC) after exposition of erythrocyte-derived microvesicles from control mice (*Jak2^WT^*) (*n* = 4) and *Jak2^V617F^ HC-EC* mice and without (red, *n* = 4) and with (*n* = 4) preincubation with an MPOi (PF06281355, 5 mol/L). (**G**) Representative images of HUVEC staining with CellROX (red fluorogenic probes for ROS generation) and DAPI (nuclei in blue). Scale bars: 10 μm. *P < 0.05, ***P < 0.001. Quantitative data are expressed as median with IQR and compared using the Mann-Whitney *U* test and Kruskal-Wallis test for multiple comparisons. CY24A, cytochrome *b*-245 light chain; CY24B, cytochrome *b*-245 heavy chain.

To shed light on the mechanisms underlying the increased oxidative stress induced by microvesicles derived from JAK2^V617F^ erythrocytes, we performed proteomic analysis of these microvesicles (data are available via ProteomeXchange; see Methods). All proteins involved in ROS detoxification or generation are shown in the volcano plot in Figure 5A, and their role in oxidative stress is summarized in Supplemental Table 1. We found two proteins that could explain the observed effect, one significantly deregulated (glutathione *S* transferase θ 1 [GSTT1]) and one detected only in microvesicles derived from JAK2^V617F^ erythrocytes (myeloperoxidase [MPO]) (Figure 5A). We also considered cytochrome *b*-245 heavy and light chain (NOX2), although only a few peptides were detected in microvesicles derived from JAK2^V617F^ erythrocytes (1 peptide of heavy chain in 1 of 6 samples and 1 peptide of light chain in 2 of 6 samples), since NOX plays a key role in oxidative stress and since no NOX2 peptide was detected in microvesicles derived from JAK2^WT^ erythrocytes. Western blot analyses were then performed on microvesicles derived from JAK2^V617F^ and control erythrocytes to verify proteomics results. By Western blot, NOX2 expression evaluated by Gp91 was not significantly different between JAK2^V617F^ and control erythrocyte-derived microvesicles (Supplemental Figure 3). Levels of GSTT1, which has an antioxidant effect (21), was significantly lower in JAK2^V617F^ than in control erythrocyte–derived microvesicles (Figure 5, B and C). Expression of MPO, a protein with a strong prooxidant effect (22), was much higher in JAK2^V617F^ than in control erythrocyte–derived microvesicles (Figure 5, D and E). We then directly inhibited MPO in microvesicles derived from erythrocytes of *Jak2^V617F^ HC-EC* mice before incubation with endothelial cells (HUVECs) in vitro. We observed that the MPO inhibitor PF06281355 completely reversed the increase in endothelial oxidative stress induced by *Jak2^V617F^* erythrocyte–derived microvesicles (Figure 5, F and G).

In conclusion, JAK2^V617F^ erythrocyte–derived microvesicles carry MPO, which confers a prooxidant phenotype in endothelial cells, leading to the increased arterial contraction observed in *Jak2^V617F^ HC-EC* mice.

### Statins as a potential new treatment in myeloproliferative neoplasms.

**Figure 6:**
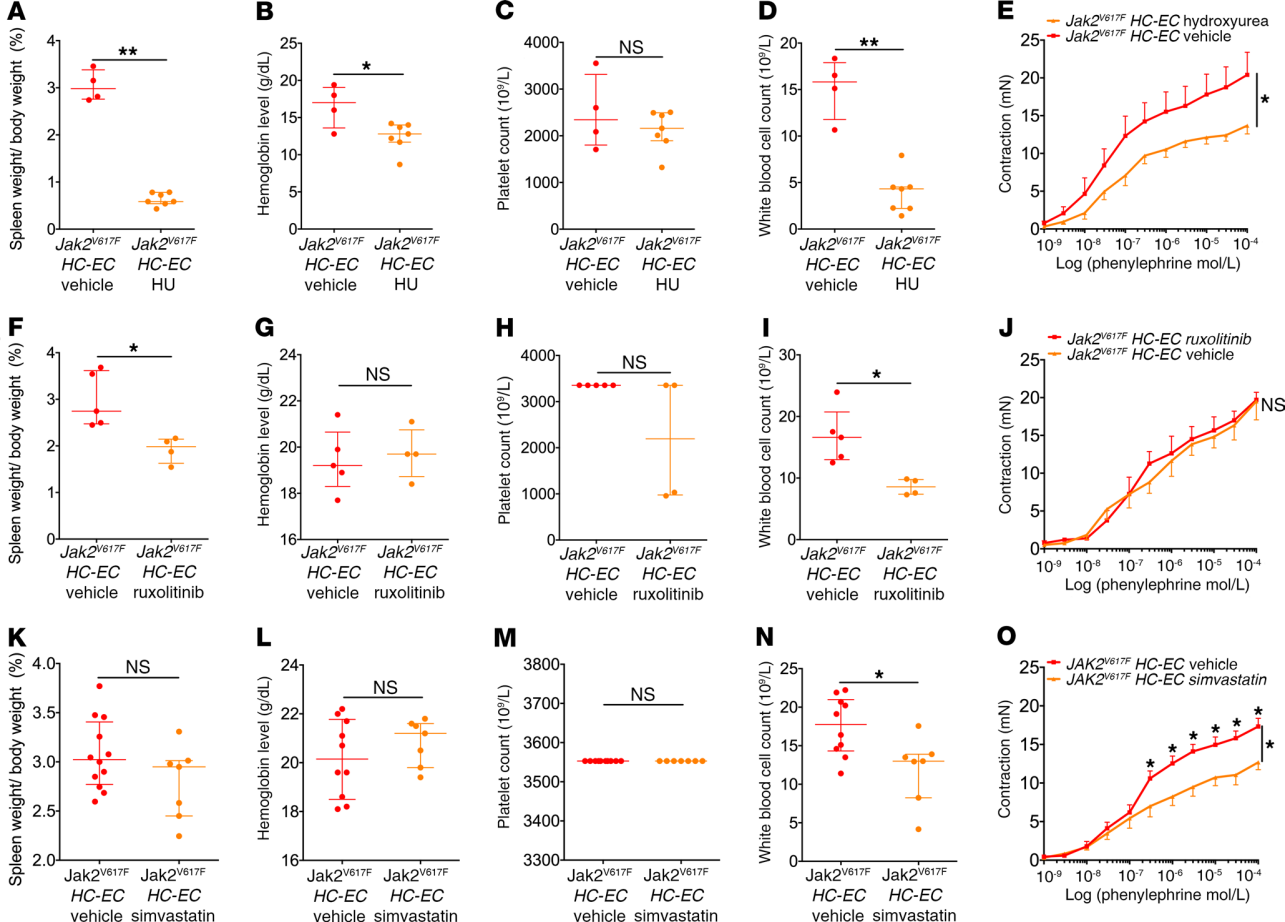
Simvastatin improves the increased arterial contraction induced by JAK2^V617F^. Spleen to body weight ratio (**A**), hemoglobin level (**B**), platelet count (**C**), and WBC count (**D**) in *Jak2^V617F^ HC-EC* mice treated with vehicle (*n* = 4) or hydroxyurea (HU, *n* = 7). Spleen to body weight ratio (**F**), hemoglobin level (**G**), platelet count (**H**), and WBC count (**I**) in *Jak2^V617F^ HC-EC* mice treated with vehicle (*n* = 5) or ruxolitinib (*n* = 4). Spleen to body weight ratio (**K**), hemoglobin level (**L**), platelet count (**M**), and WBC count (**N**) in *Jak2^V617F^ HC-EC* mice treated with vehicle (*n* = 10) or simvastatin (*n* = 7). Cumulative dose-response curves to phenylephrine of aortas from *Jak2^V617F^ HC-EC* mice treated with vehicle or hydroxyurea (*n* = 4 and *n* = 7) (**E**), with vehicle or ruxolitinib (*n* = 5 and *n* = 4) (**J**), and with vehicle or simvastatin (*n* = 10 and *n* = 7) (**O**). *P < 0.05, **P < 0.01. Data are expressed as mean with SEM for cumulative curves and median with IQR for spleen weight and blood cell counts. Cumulative dose-response curves were compared using ANOVA for repeated measures, and other data were compared using the Mann-Whitney *U* test. All tests were 2 sided.

We then tested whether available treatments for MPNs, namely hydroxyurea and ruxolitinib, affect this increased arterial contraction. In *Jak2^V617F^ HC-EC* mice, which best represent the human disease, hydroxyurea treatment for 10 consecutive days decreased spleen weight, hemoglobin level, and WBC count (Figure 6, A, B, and D). However, platelet count was not affected by this short-duration hydroxyurea treatment (Figure 6C). Hydroxyurea significantly improved contraction in response to phenylephrine as compared with vehicle (Figure 6E).

We then treated *Jak2^V617F^ HC-EC* mice with ruxolitinib for 21 consecutive days and observed a significant decrease in spleen weight and WBC count (Figure 6, F and I), but no effect on hemoglobin level or platelet count (Figure 6, G and H). Ruxolitinib had no effect on arterial response to phenylephrine (Figure 6J).

Beyond its cholesterol-lowering effect, simvastatin also improves endothelial function through the NO pathway and by preventing oxidative stress damage (23, 24). Thus, we tested its effect on the arterial response to phenylephrine in *Jak2^V617F^ HC-EC* mice. Fourteen days of treatment with simvastatin did not change spleen weight, hemoglobin level, or platelet count (Figure 6, K–M). There was only a slight decrease in WBC count following simvastatin treatment (Figure 6N). Interestingly, simvastatin significantly improved the aortic response to phenylephrine as compared with vehicle (Figure 6O).

## Discussion

This study demonstrated that JAK2^V617F^ erythrocyte–derived microvesicles carrying MPO are responsible for increased oxidative stress in arterial endothelium and decreased availability of NO, which strongly increased arterial contraction in response to vasoconstrictive agents, possibly accounting for the arterial events associated with MPNs. Simvastatin, a drug with antioxidant properties, improved arterial contraction.

The first major finding of our study is that *JAK2^V617F^* MPN induces a considerable increase in arterial contraction. This finding suggests a vasospastic phenomenon associated with MPN and thus represents a paradigm shift with regard to MPNs, as arterial events have only been seen to be a result of a thrombotic process (7). Our results obtained ex vivo and in vivo could explain this higher incidence of arterial events in patients with polycythemia vera than in the general population and the high prevalence of myocardial infarction without significant coronary stenosis by angiography in patients with MPN (8). Arterial spasm is an underdiagnosed phenomenon that can occur in patients without atherosclerosis, but underlying nonstenotic atherosclerotic plaques are also known to be a contributing factor. This suggests that the effect we observed might account not only for the myocardial infarction without significant coronary stenosis reported in patients with MPN, but might also more widely for arterial events in patients with atherosclerotic plaques and MPN. Moreover, arterial spasm occurs not only in coronary arteries, but also in brain arteries (25). We also found an impairment in arterial dilatation, which is in line with the altered endothelium-dependant, flow-mediated vasodilatation reported in patients with polycythemia vera in the absence of overt arterial disease (26).

The second major finding of our work is the contribution of JAK2^V617F^ erythrocyte–derived microvesicles to this increased arterial contraction associated with MPN. Importantly, we observed this effect with JAK2^V617F^ erythrocyte microvesicles from mice as well as microvesicles isolated from patients carrying JAK2^V617F^. We thus highlight here a crucial vascular role for microvesicles in MPNs, beyond their already described involvement in coagulation in this setting (27–31). Although patients with MPNs have higher circulating levels of microvesicles than healthy individuals, we assessed vascular reactivity using the same concentrations of microvesicles for both groups, which suggests that microvesicle composition, and not concentration, accounts for the observed vascular effect (27, 28, 32–35). We cannot rule out the possibility that JAK2^V617F^ erythrocytes themselves, in addition to microvesicles, also directly increase arterial contraction associated with MPNs.

Finally, we demonstrated that NO pathway inhibition and increased endothelial oxidative stress are implicated in this increased arterial contraction associated with MPN. Several groups have reported high levels of circulating ROS products (36–38) and low antioxidant status in MPN (37, 39), but endothelial oxidative stress has not to our knowledge been investigated. Erythrocyte microvesicles have already been linked to vascular dysfunction in various settings, such as erythrocyte storage and sickle cell disease (18, 19, 40), but not in the context of MPN. Thanks to proteomics approaches, we were able to identify a defect in GSTT1 and overexpression of MPO in microvesicles derived from JAK2^V617F^ erythrocytes. Using direct and irreversible inhibition of MPO, we were able to ascertain the role of MPO carried by microvesicles derived from JAK2^V617F^ erythrocytes in the increase in endothelial oxidative stress. MPO is a polycationic heme-containing glycoprotein stored mainly in the azurophilic granules of neutrophils, but up to 30% of total cellular MPO can be released as active enzyme into the extracellular space. Interestingly, extracellular MPO can bind to the RBC membrane and is associated with endothelial dysfunction in the context of ischemic heart disease (41–45). Our results demonstrate that MPO binds to erythrocyte-derived microvesicles, and increases endothelial oxidative stress and the vascular response to vasoconstrictors.

The role of GSTT1 in the increased endothelial oxidative stress status and vascular reactivity we observed with microvesicles derived from JAK2^V617F^ erythrocytes remains uncertain, because we could not restore a normal level of GSTT1 only in microvesicles. The normalization of vascular reactivity induced by NAC could be explained by the glutathione inducer activity of NAC, but could also just be due to the potent antioxidant activity of this drug.

In addition to demonstrating how *JAK2^V617F^* induces this increased arterial contraction, our results open potential therapeutic perspectives to prevent cardiovascular events in patients with MPN. We demonstrated that simvastatin, a well-known and easily accessible drug, strongly improved the arterial response to a vasoconstrictive agent in our MPN mouse model. These results thus pave the way for testing the use of simvastatin to prevent arterial events in patients with MPN. We also tested available treatments for MPN and observed that hydroxyurea, but not ruxolitinib, improved arterial contraction. This difference might be explained by the fact that hydroxyurea decreased erythrocyte counts in our mouse model, whereas ruxolitinib did not (46, 47).

In conclusion, our study showed that microvesicles derived from erythrocytes are responsible for increased arterial contraction in *JAK2^V617F^* MPNs. This effect is due to overexpression of MPO in JAK2^V617F^ erythrocyte–derived microvesicles, which is responsible for increased endothelial oxidative stress and NO pathway inhibition. Simvastatin appears to represent a new treatment approach to prevent arterial events in MPN, which warrants further study.

## Methods

### Experimental design.

The objective of our study was to analyze endothelial reactivity in MPN. We first noticed a substantial increase in arterial contraction in *Jak2^V617F^ HC-EC* mice, a model with *Jak2^V617F^* expression in both hematopoietic and endothelial cells that mimics the human disease. We created mouse models specifically mutated in endothelial or hematopoietic cells. We then searched for the mediators responsible for the increased response to vasoconstrictors when *Jak2^V617F^* was present in hematopoietic cells; we tested the hypothesis that circulating blood might convey biological information from hematopoietic cells to the vascular wall, and focused on microvesicles. We identified that erythrocyte-derived microvesicles were responsible for this effect and performed mass spectrography analysis to identify the proteins involved. Sample size was chosen based on previous works using the same technique (myography) and microvesicles published by our team (48, 49).

Mouse breeding occurred in our animal facility in accordance with local recommendations. Control mice were matched with littermates of the appropriate, age, sex, and genetic background to account for any variation in data.

Numbers of experimental replicates are shown in the figure legends, and at least 3 independent experiments were performed. For each myography experiment, duplicates with the same aorta were used, averaged, and counted as *n* = 1. There was no randomization in these experiments. We did not exclude any samples other than those not fulfilling the quality criteria detailed in *Organ chamber experiments*. Indeed, only aortas with a viable endothelium were used for myography (i.e., relaxation to acetylcholine ≥70% of the precontraction).

For human samples, inclusion and exclusion criteria were defined before sample collection (see *Patient inclusion*). No outlier was excluded. Investigators were not blinded to group allocation during collection and analysis of the data. Donors included patients carrying *JAK2^V617F^* with a past history of splanchnic vein thrombosis who had not received any specific treatments other than vitamin K antagonists, and healthy volunteers.

### Murine models.

All mice were on a C57BL/6 background. Mice carrying a constitutive *Jak2^V617F^* mutation in endothelial and hematopoietic cells were obtained by crossing *VE-cadherin-Cre* transgenic mice (13) with *Jak2^V617F^^Flex/WT^* mice (50) generated by our research team. Mice carrying the inducible *JAK2^V617F^* mutation specifically in endothelial cells were obtained by crossing *VE-cadherin-Cre-ERT2* transgenic mice provided by R.H. Adams (Max Planck Institute for Molecular Biomedicine, Münster, Germany) (51) with *Jak2^V617F^^Flex/WT^* mice generated in-house (50). The Flex (Flip-Excision) strategy allows expression of a mutated gene in adulthood, in a temporal and tissue-specific manner (52). It allows an efficient and reliable Cre-mediated genetic switch: the expression of a given gene is turned on by inversion, while expression of another one is simultaneously turned off by excision. In all experiments, male and female mice were used.

For organ chamber experiments and femoral in vivo experiments, mice were euthanized between the ages of 8 and 17 weeks. For induction of the Cre recombinase expression in *Jak2^V617F Flex/WT^ VE-cadherin-Cre-ERT2* mice, mice were injected intraperitoneally with 1 mg/mouse/d tamoxifen (Sigma-Aldrich, T5648) for 5 consecutive days over 2 consecutive weeks (10 mg in total per mouse) between the ages of 5 and 7 weeks. Experiments were performed between 4 and 6 weeks after the last tamoxifen injection. Both female and male mice were used for each experiment.

### Verification of the efficient endothelial recombination in mouse models.

All mice were on a C57BL/6 background. Mice with the *mTmG* reporter were crossed with *VE-cadherin-Cre* transgenic mice (both generated in-house) or *VE-cadherin-Cre-ERT2* transgenic mice provided by R.H. Adams (51). For induction of the *mTmG VE-cadherin-Cre-ERT2* model, mice were injected intraperitoneally with 1 mg/mouse/d tamoxifen (Sigma-Aldrich, T5648) for 5 consecutive days over 2 consecutive weeks (10 mg in total per mice) between the ages of 5 and 7 weeks, and experiments were performed 2 weeks after the last injection of tamoxifen. Aortas and femurs were harvested under isoflurane anesthesia and fixed in 4% PFA. Aortas were mounted en face on glass slides, while femurs were cryosectioned. All tissues were imaged using a Leica SP5 confocal microscope at ×400 magnification. For flow cytometry analysis in *mTmG VE-cadherin-Cre-ERT2* mice, bone marrow cells were stained with Ter-119 APC and Gr-1 APC (BD Biosciences, 553673) and analyzed on an Accuri C6 flow cytometer (BD Biosciences). Data were interpreted using BD Accuri C6 Software.

### Patient inclusion.

All patients fulfilling inclusion criteria were prospectively included at the Hepatology Department, Beaujon Hospital, Clichy, France, between May and July 2016. Only patients carrying *JAK2^V617F^* without specific treatment for MPNs were included. All patients had a history of Budd-Chiari syndrome or portal vein thrombosis and were receiving vitamin K antagonists. Controls were healthy volunteers.

### Organ chamber experiments.

Thoracic aortas from adult mice were isolated after animal sacrifice under 2% isoflurane anesthesia. The aortic rings were mounted immediately in organ chambers (Multi Wire Myograph System, DMT, model 610M) filled with Krebs-Ringer solution ( 118.3 mmol/L NaCl, 4.7 mmol/L KCl, 1.2 mmol/L MgSO_4_, 1.2 mmol/L KH_2_PO_4_, 1.25 mmol/L CaCl_2_, 25.0 mmol/L NaHCO_3_, 5.0 mmol/L, and glucose) and gassed with a mixture of 95% O_2_ and 5% CO_2_ (pH 7.4). The presence of functional endothelial cells was confirmed by relaxation to acetylcholine chloride (Sigma-Aldrich, A6625) (10^–5^ mol/L) following a contraction evoked by phenylephrine (10^–7^ mol/L), defined as a relaxation ≥70% of the precontraction as previously described (49). After extensive washout and equilibration, contraction to phenylephrine hydrochloride (concentration-response curve, 10^–9^ to 10^–4^ mol/L) (Sigma-Aldrich, P1250000), angiotensin II (concentration-response curve, 10^−9^ to 10^−6^ mol/L) (Sigma-Aldrich, A9525), or KCl (80 mmol/L) and relaxation to acetylcholine chloride (concentration-response curve, 10^−9^ to 10^−4^ mol/L) or SNAP (Sigma-Aldrich, N3398) (concentration-response curve, 10^−10^ to 10^−5^ mol/L) was studied. For NO synthase inhibition, aorta rings were preincubated for 45 minutes with 10^–4^ mol/L l-NAME (Cayman, 80210) before concentration-response curve to phenylephrine without washout were performed. In some experiments, the endothelium was mechanically removed by inserting the tip of forceps within the lumen and gently rubbing the ring back and forth on a piece of wet tissue. For the NAC experiment (commercial HIDONAC, Zambon), NAC was added to the Krebs-Ringer solution at a final concentration of 20 mmol/L.

### In vivo femoral reactivity.

The femoral artery of adult mice was exposed under 2% isoflurane anesthesia. Krebs-Ringer solution (see *Organ chamber experiments*) gassed with a mixture of 95% O_2_and 5% CO_2_ (pH 7.4) at 37°C was permanently superfused (2 mL/min) on the exposed artery. After a 15-minute equilibration, arterial responses were determined by addition of phenylephrine (10^−3^ mol/L) for 2 minutes, then acetylcholine (10^−1^ mol/L) for 1 minute (Sigma-Aldrich). On the contralateral leg, with the same protocol, KCl (80 mmol/L) and then SNAP (10^−3^ mol/L) were used. All dilutions were prepared just before application. Changes in vessel diameter were continuously recorded on a videotape recorder. Subsequently, images were exported and vessel outer diameters analyzed using ImageJ (NIH) software.

### Isolation and characterization of patients’ circulating microvesicles.

Circulating microvesicles from patients or healthy control individuals were isolated from platelet-free plasma obtained by successive centrifugations of venous blood, as reported previously (53). Briefly, citrated venous blood (15 mL) was centrifuged twice at 2500 *g* for 15 minutes (at room temperature) to remove cells and cell debris and to obtain platelet-free plasma (PFP). A portion of this PFP was then aliquoted and stored at –80°C. The rest was centrifuged at 20,500 *g* for 2 hours (4°C). Supernatant of this 20,500 *g* centrifugation was then discarded, and the resulting microvesicles pellet was resuspended in a minimal volume of supernatant, aliquoted, and stored at –80°C. For each patient, concentrations of annexin V–positive microvesicles were analyzed in the PFP and the resuspended pellet of microvesicles.

Circulating levels of annexin V–positive microvesicles (Beckman Coulter, IM3614) were determined on a Gallios flow cytometer (Beckman Coulter) using a technique previously described in detail (49, 53).

### Generation of microvesicles from mice.

Blood samples were collected from the inferior vena cava of *Jak2^V617F^ HC-EC* mice or littermate controls using a 25 G × 1 inch needle in a 1-mL syringe precoated with 3.8% sodium citrate. PFPs were generated as described above for patients and used to measure plasma annexin V–positive microvesicles in mice. The pelleted cells obtained following the first 2500-*g* centrifugation were resuspended in PBS to a final volume of 5 mL for control mice and 10 mL for *Jak2^V617F^ HC-EC* mice. PBMCs, PMNCs, and erythrocytes were separated using a double Percoll gradient (63% and 72% for control mice, and 63% and 66% for *Jak2^V617F^ HC-EC* mice) using a 700-*g* centrifugation for 25 minutes, without braking. The slight differences between the protocols used for control and *Jak2^V617F^ HC-EC* mice were the result of the preliminary experiments we performed to obtain pure isolation of each cell type. Cells were subsequently washed with PBS, then incubated with 5 μmol/L ionomycin in TBS for 30 minutes at 37°C to induce microvesicle generation. 5 mmol/L EDTA was added to chelate free calcium. Cells were then discarded by centrifugations at 15,000 *g* for 1 minute, and the supernatants were collected. Microvesicles were isolated as described above using a 20,500-*g* centrifugation during 45 minutes. Concentrations of annexin V–positive microvesicles (as described above) were analyzed in the PFP and the 20,500-*g* microvesicle pellet for each mouse.

To isolate platelets, 500 μL of whole blood was diluted in 10 mL PBS. A 1.063-g/mL density barrier was created by combining 5 mL of 1.320 g/mL 60% iodixanol stock solution (OptiPrep density gradient medium, Sigma-Aldrich) with 22 mL diluent (0.85% NaCl, 20 mM HEPES-NaOH, pH 7.4, 1 mM EDTA). For platelet separation, 10 mL diluted blood from control and *Jak2^V617F^ HC-EC* mice was layered over a 10-mL density barrier and centrifuged at 350 *g* for 15 minutes at 20°C with the brake turned off. The interface between the density barrier and the blood contained platelets. Residual contaminating erythrocytes were removed by magnetic sorting. Briefly, the cell suspension was labeled with Anti-Ter-119 MicroBeads (Miltenyi Biotec, 130-049-901), and erythrocytes (Ter-119–positive) were negatively sorted using a MACS Separator. The remaining cells (platelets) were subsequently washed with PBS and exposed to 5 μmol/L ionomycin in TBS for 30 minutes at 37°C. 5 mM EDTA was then added to chelate free calcium. Finally, cells were discarded by centrifugation at 15,000 *g* for 1 minute, the supernatant was collected, and microvesicles were isolated, as described above.

### Vascular reactivity following exposure to microvesicles.

For organ chamber experiments, thoracic aortas from adult C57BL/6 mice (8–10 weeks old) were isolated after sacrifice under isoflurane anesthesia. Mouse aortic rings were incubated for 24 hours at 37°C in a 5% CO_2_ incubator, with filtered DMEM supplemented with antibiotics (100 IU/mL streptomycin, 100 IU/mL penicillin [Gibco, Invitrogen], and 10 μg/mL polymyxin B [Sigma-Aldrich]) in the presence of microvesicles. Aortic rings were then mounted in organ chambers, and concentration-response curves to pharmacological agents were performed.

For in vivo femoral artery experiments, C57BL/6 mice were injected intravenously (retro-orbital injection) with microvesicles (100 μL final volume with 2 μL heparin sodium [5000 IU/mL]). Experiments were performed 2 hours after injection.

Microvesicles from patients and healthy controls were incubated at their respective individual plasma concentration (annexin V–positive microvesicles). Microvesicles generated from mice were incubated or injected at the same final concentration as for *Jak2^V617F^ HC-EC* mice and control mice, namely 7000 annexin V–positive microvesicles/μL for erythrocyte- and platelet-derived microvesicles and 700 annexin V–positive microvesicles/μL for PBMC- and PMNC-derived microvesicles. We chose these concentrations because we found in preliminary experiments that the majority of mice had concentrations of annexin V–positive microvesicles between 1000 and 10,000/μL, and because PBMC- and PMNC-derived microvesicles are consistently found to be less abundant in the blood than erythrocyte- and platelet-derived microvesicles (27, 32).

### Bone marrow transplantation.

We subjected 6- to 8-week-old C57BL/6J mice to medullar aplasia following lethal 9.5-Gy total body irradiation. We repopulated the mice with an intravenous injection of bone marrow cells isolated from femurs and tibiae of age-matched *Jak2^V617F^ HC-EC* and littermate control mice. Medullar reconstitution was allowed for 8 weeks before experiments were performed.

### Treatments.

Hydroxyurea (Sigma-Aldrich, H8627) or the same volume of vehicle (0.9% NaCl) was administrated for 10 consecutive days (100 mg/kg/d bid) by intraperitoneal injections.

Ruxolitinib (Jakavi, Novartis) was administered for 21 consecutive days (30 mg/kg, 2 times per day) by oral gavage (54). Ruxolitinib was prepared from 15-mg commercial tablets in PEG300/5% dextrose mixed at a 1:3 ratio, as previously reported (55). Control mice were administered the same volume of vehicle (PEG300/5% dextrose).

Simvastatin (Sigma-Aldrich, S6196) was administered for 14 days (20 mg/kg/d, once a day) by intraperitoneal injections. Activation by hydrolysis was first achieved by dissolving 50 mg in 1 mL pure ethanol and adding 0.813 mL of 1 mol/L NaOH. pH was adjusted to 7.2 by adding small quantities of 1 mol/L HCl, and dilution was then performed in PBS (56). Control mice were injected with the same volume of vehicle.

Human recombinant epoetin alfa (5000 UI/kg, diluted in 0.2% BSA in PBS) or vehicle (0.2% BSA in PBS) was administered to WT mice every 2 days for 3 weeks by intraperitoneal injection, as previously described (57).

NAC (HIDONAC) diluted in 0.9% NaCl or the same volume of vehicle (0.9% NaCl) was administrated for 14 consecutive days (500 mg/kg/d) by intraperitoneal injections.

### Blood cell count analysis.

Blood was collected on the day of sacrifice from the inferior vena cava using a 25G × 1 inch needle in a 1-mL syringe precoated with 3.8% sodium citrate. Blood count analyses were performed using a Hemavet 950FS analyzer (Drew Scientific).

### Quantification of ROS generation.

Thoracic aortas from adult mice were isolated after animal sacrifice under 2% isoflurane anesthesia, longitudinally opened, and placed directly in HBSS (Sigma-Aldrich, 14025-092). For each set of experiments, all aortas were processed immediately after removal, at the same time, with the same reagents, and in the same manner. No plasma factor or blood cells were added during the ROS generation assessment. For positive and negative controls, 2 pieces of WT aortas were incubated with H_2_O_2_ (100 μmol/L final concentration) for 20 minutes at 37°C. For negative controls, NAC (5 mmol/L final concentration) was incubated together with H_2_O_2_ for 20 minutes at 37°C. All aortas were then incubated with 5 μmol/L CellROX (Thermo Fisher Scientific, C10422) for 30 minutes at 37°C. CellROX Deep Red Reagent is a fluorogenic probe designed to reliably measure ROS inside living cells. The cell-permeable CellROX Deep Red dye is nonfluorescent while outside the cell and in a reduced state and upon oxidation exhibits excitation/emission maxima at 640/665 nm. After rinsing and fixation (4% paraformaldehyde, 20 minutes), samples were costained with DAPI (0.1 μg/mL, Sigma-Aldrich) in order to identify cell nuclei. After staining, aortas were washed with PBS, mounted en face on glass slides, and imaged using a bright-field Axio Imager Z1 (Zeiss) microscope. Images were acquired in the 2 hours following staining at ×400 magnification. CellROX-positive surface (in red) and cell numbers were quantified using ImageJ software.

### MPO inhibition in microvesicles.

Erythrocyte-derived microvesicles from *Jak2^V617F^ HC-EC* mice were incubated for 1 hour with an irreversible MPO inhibitor (MPOi, PF06281355, resuspended in DMSO, Sigma-Aldrich) diluted in PBS (5 mol/L final concentration). Then, the same amount of annexin V–positive erythrocyte-derived microvesicles (JAK2^WT^, JAK2^V617F^, and JAK2^V617F^ with MPOi) were washed in PBS and centrifuged at 20,500 *g* for 2 hours. The pellet containing the microvesicles was then resuspended in endothelial cell basic medium (PromoCell). HUVECs (single donor; PromoCell, C-12200, lot 445Z011) were then incubated for 2 hours at 37°C with these microvesicles. At the end of the incubation, and without washing cells, ROS generation was assessed using CellROX, as described above. After rinsing with medium and paraformaldehyde (4%, 5 minutes), HUVECs were costained with DAPI (0.1 μg/mL, Sigma-Aldrich) in order to identify nuclei. Images were acquired using a Leica SP8 confocal microscope at ×400 magnification.

### Electrocardiography.

Electrocardiograms were recorded from mice using noninvasive ecgTUNNEL (Emka Technologies) with minimal filtering. ECG signal was continuously monitored for 3 minutes (baseline). Waveforms were recorded using Iox Software, and heart rate and intervals were measured with ecgAUTO from recording traces. Following baseline determination, the animals received a single administration of phenylephrine (bolus, 3 mg/kg) by the intravenous route at the caudal vein, and electrocardiograms were recorded 3–5 minutes more.

### RNA gene allelic discrimination.

Erythrocyte microvesicles were lysed with QIAzol lysis reagent (QIAGEN), and RNA was extracted with an RNeasy Micro Kit (QIAGEN) according to the manufacturer’s instructions. RNA was quantified with a Qubit RNA HS Assay Kit (Thermo Fisher Scientific). cDNA synthesis was performed with a QuantiTect Reverse Transcription Kit (QIAGEN). RNA gene allelic discrimination was performed by TaqMan analysis with the ABI Prism GeneAmp 7500 Sequence Detection System (Applied Biosystems, Invitrogen) using the following as primers: TTTACAAATTCTTGAACCAGAATGTTC (JAK2 forward) and TTCTCACAAGCATTTGGTTTTGAAT (JAK2 reverse); and as probes: VIC-CTCCACAGACACAGAC-MGB for *JAK2^WT^* and 6-FAM-TCTCCACAGAAACAGAGA-MGB for *JAK2^V617F^*.

### Mass spectrometry analysis.

Size-exclusion chromatography of microvesicles was then performed in order to separate microvesicles from soluble proteins. Successive aliquot of 150 μL were collected, and measurement of protein absorbance was performed. Fractions contained in tubes 6–11, containing microvesicles, were selected and then centrifuged at 20–500 *g* for 2 hours. To finish, microvesicles were lysed using 1% Triton buffer.

For mass spectrometry analysis, proteins were precipitated overnight at –20°C with 0.1 mol/L ammonium acetate glacial in 80% methanol (buffer 1). After centrifugation at 14,000 *g* and 4°C for 15 minutes, the resulting pellets were washed twice with 100 μL buffer 1 and further dried under vacuum (Savant Centrifuge SpeedVac concentrator, Thermo Fisher Scientific). Proteins were then reduced by incubation with 10 μL of 5 mmol/L DTT at 57°C for 1 hour and alkylated with 2 μL of 55 mmol/L iodoacetamide for 30 minutes at room temperature in the dark. Trypsin/LysC (Promega) was added twice at 1:100 (wt/wt) enzyme/substrate, at 37°C for 2 hours first and then overnight. Samples were then loaded onto a homemade C18 StageTips for desalting. Peptides were eluted using 40:60 MeCN/H_2_O plus 0.1% formic acid and vacuum concentrated to dryness. Online chromatography was performed with an RSLCnano system (Ultimate 3000, Thermo Fisher Scientific) coupled online to a Q Exactive HF-X with a Nanospray Flex ion source (Thermo Fisher Scientific). Peptides were first trapped on a C18 column (75-μm inner diameter × 2 cm; nanoViper Acclaim PepMap 100, Thermo Fisher Scientific) with buffer A (2:98 MeCN/H_2_O in 0.1% formic acid) at a flow rate of 2.5 μL/min over 4 minutes. Separation was then performed on a 50 cm × 75 μm C18 column (nanoViper Acclaim PepMap RSLC, 2 μm, 100 Å) regulated to a temperature of 50°C with a linear gradient of 2%–30% buffer B (100% MeCN in 0.1% formic acid) at a flow rate of 300 nL/min over 91 minutes. MS full scans were performed in the ultrahigh-field Orbitrap mass analyzer in the *m*/*z* range of 375–1500 with a resolution of 120,000 at *m*/*z* 200. The 20 most intense ions were subjected to Orbitrap for further fragmentation via high-energy collision dissociation (HCD) activation and a resolution of 15,000, with the intensity threshold kept at 1.3 × 10^5^. We selected ions with charge state from 2+ to 6+ for screening. Normalized collision energy (NCE) was set at 27 and a dynamic exclusion of 40 seconds.

For identification, data were searched against the *Mus musculus* one gene one protein (UP000000589_10090) UniProt database and a databank of the common contaminants using SEQUEST HT through Proteome Discoverer (version 2.2, Thermo Fisher Scientific). Enzyme specificity was set to trypsin, and a maximum of 2 missed cleavage sites were allowed. Oxidized methionine and N-terminal acetylation were set as variable modifications. Maximum allowed mass deviation was set to 10 ppm for monoisotopic precursor ions and 0.02 Da for MS/MS peaks. The resulting files were further processed using myProMS (58) v3.6. FDR calculation used Percolator (59) and was set to 1% at the peptide level for the whole study. The label-free quantification was performed by peptide extracted ion chromatograms (XICs) computed with MassChroQ version 2.2 (60). For protein quantification, XICs from proteotypic peptides shared by compared conditions (TopN matching) with 2 missed cleavages were used. Median and scale normalization was applied on the total signal to correct the XICs for each biological replicate. To estimate the significance of the change in protein abundance, a linear model (adjusted on peptides and biological replicates) was performed, and *P* values were adjusted with a Benjamini-Hochberg FDR procedure with a control threshold set to 0.05.

The mass spectrometry proteomics data were deposited into ProteomeXchange via the PRIDE database (61) partner repository (data set identifier PXD014451).

### Western blot on erythrocyte-derived microvesicles.

Erythrocyte-derived microvesicles generated as mentioned above were centrifuged at 20,500 *g* for 2 hours and then lysed in 100 μL RIPA buffer containing 150 mmol/L NaCl, 50 mmol/L Tris-HCl, pH 7.4, 2 mmol/L EDTA, 0.5% sodium deoxycholate, 0.2% SDS, 2 mmol/L activated orthovanadate, complete protease inhibitor cocktail tablet (Complete Mini, Roche), and complete phosphatase inhibitor cocktail tablet (Roche). Protein content was quantified using the Micro BCA Protein Assay Kit (Thermo Fisher Scientific). Equal loading was checked using Ponceau red solution. Membranes were incubated with primary antibodies (1:1000) (anti-GP91, BD, 611415; anti-GSTT1, Abcam, 199337; anti-MPO, Abcam, 45977). After secondary antibody incubation (anti-rat, Cell Signaling Technology, 1:1000; anti-rabbit or anti-mouse, Amersham, GE Healthcare, 1:3000), immunodetection was performed using an enhanced chemiluminescence kit (Immun-Star WesternC kit, Bio-Rad). Bands were revealed using the LAS-4000 imaging system (GE Healthcare Life Sciences). Values reported from Western blots were obtained by band density analysis with ImageJ software and expressed as the ratio of protein of interest to Ponceau.

### Uptake of microvesicles by endothelial cells.

Erythrocyte-derived microvesicles were stained with PKH26 dye (Sigma-Aldrich) diluted in PBS following the manufacturer’s instructions, washed in PBS, and then centrifuged at 20,500 *g* for 2 hours. The 20,500-*g* supernatant was used for control experiments. Murine endothelial cells (the cell line SVEC4-10, CRL-2181, ATCC, lot 70008729) were then incubated with these stained microvesicles or an equal volume of the 20,500-*g* supernatant. After 2 hours at 37°C, cells were washed 3 times with DMEM (Gibco). Cells were then fixed in 4% PFA for 5 minutes and then costained with DAPI (0.1 g/mL, Sigma-Aldrich) in order to identify cell nuclei. Images were acquired using a Leica SP8 confocal microscope at ×600 magnification.

### Statistics.

For cumulative dose-response curves, data are expressed as mean with SEM and were compared using an ANOVA for repeated measures. Other data are expressed as median with IQR (blood cell count and spleen weight) and were compared using the Mann-Whitney *U* test. All tests were 2 sided, and differences were considered significant when *P* was less than 0.05. Data handling and analysis were performed with GraphPad Prism Software.

### Study approval.

Experiments were conducted according to French veterinary guidelines and those formulated by the European Community for experimental animal use (L358-86/609EEC), and were approved by the French Ministry of Agriculture (A75-15-32). The Institutional Animal Care and Use Committee at Inserm (Université Paris–Descartes, Paris, France, CEEA-17-053) approved all animal experiments. All donors gave written informed consent to participate in the study. Human study was performed in accordance with the ethical guidelines of the 1975 Declaration of Helsinki and was approved by the Institutional Review Board (Comités de protection des personnes [CPP], Ile de France IV, Paris, France).

## Author contributions

JP and PER designed the experiments and wrote the manuscript. CMB participated in experimental design, critically analyzed all experiments, and helped with writing of the manuscript. JP, HD, and FC performed myography experiments. JP performed oxidative stress experiments, and MT generated microvesicles. JLV, CJ, and MS provided transgenic mice. MBEM, CD, and SM characterized the first mouse model. JL managed the mouse colony. MK performed *mTmG* experiments. AP recruited the patients and healthy control individuals. TD performed JAK2^V617F^ RNA gene allelic discrimination on microvesicles. OBB analyzed heme and hemoglobin content in microvesicles. SNH and NM performed the electrocardiography analysis. DL and FD performed the mass spectrometry analysis. PER obtained funding for the project. All authors discussed and critically revised the manuscript.

## Supplementary Material

Supplemental data

## Version 1 02/11/2020

In-Press Preview

## Version 2 04/20/2020

Electronic publication

## Version 3 05/01/2020

Print issue publication
