# Supplementary material for: Erythrocyte-derived microvesicles induce arterial spasms in JAK2V617F myeloproliferative neoplasm
Source: J Clin Invest. 2020 Apr 20;130(5):2630–43. doi: 10.1172/JCI124566 (PMC7190923; doi:10.1172/JCI124566)
Supplement: Supplemental data [file jci-130-124566-s405.pdf]

Title: Erythrocyte-derived microvesicles induce arterial spasms in *JAK2^{V617F}* myeloproliferative neoplasm

Authors: Johanne Poisson[1,2,3], Marion Tanguy[1,2], Hortense Davy[1], Fatoumata Camara[1], Marie-Belle El Mdawar[1], Marouane Kheloufi[1], Cécile Devue[1], Juliette Lasselin[1], Aurélie Plessier[4,5], Salma Merchant[6], Olivier Blanc-Brude[1], Michèle Souyri[7], Nathalie Mougenot[8], Florent Dingli[9], Damarys Loew[9], Stephane N. Hatem[10], Chloé James[11,12,13], Jean-Luc Villeval[6], Chantal M. Boulanger[1], Pierre-Emmanuel Rautou[1,2,4,5]*

Supplementary Materials:
Figures S1-S4
Table S1

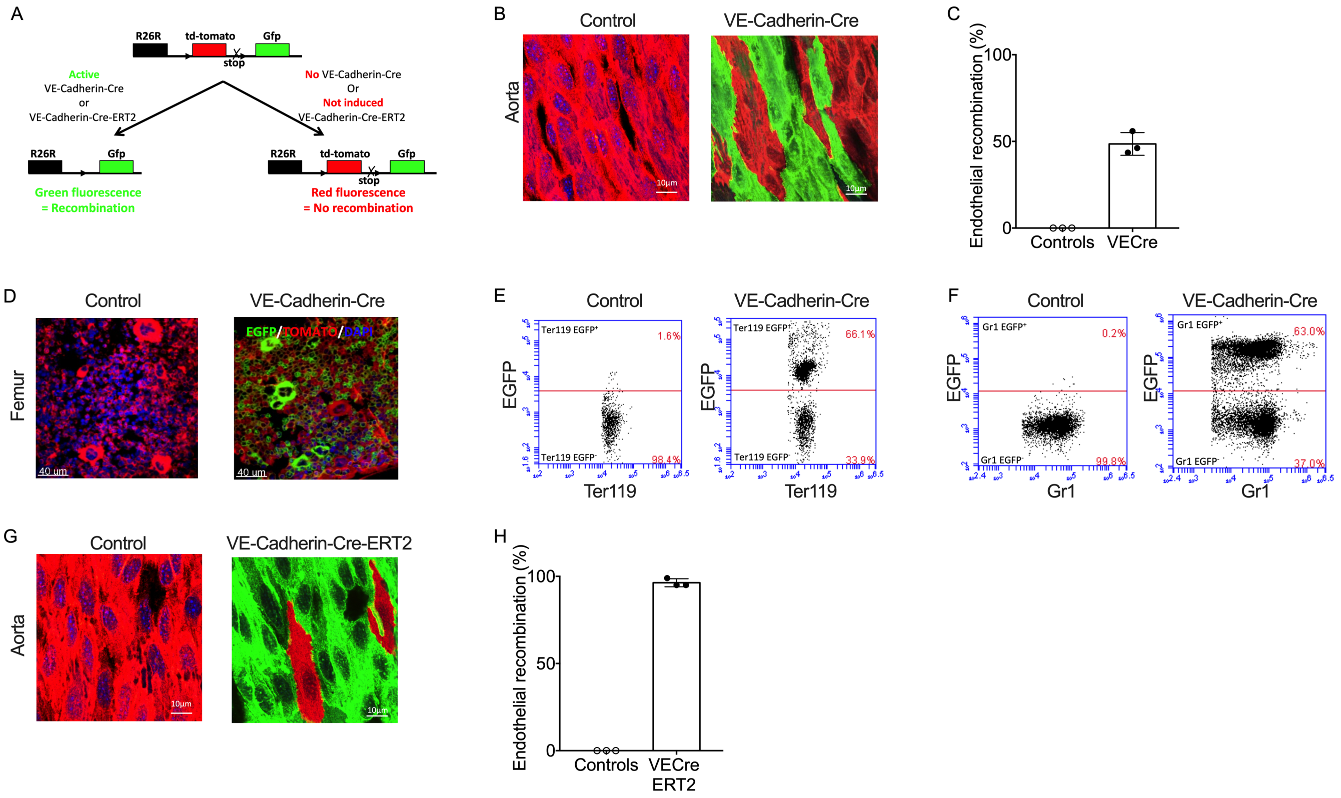

Figure *S1. Endothelial and hematopoietic progenitors' recombination.*

Mechanism of mTmG reporter strategy ***(A).*** *Representative "en face" images of the endothelium of aortas from constitutive mTmG;VE-Cadherin-Cre (n=3) mice* ***(B)*** *and inducible mTmG;VE-Cadherin-Cre-ERT2 (n=3) mice* ***(G)*** *(EGFP, green; tdTomato, red). Respective quantification* ***(C & H).*** *Confocal imaging of hematopoietic cells in adult femur sections from constitutive mTmG;VE-Cadherin-Cre mice (Nucleus, DAPI, blue; EGFP, green; tdTomato, red)* ***(D).*** *Flow cytometry analysis of hematopoietic progenitors isolated from femur bone marrow showed that the majority of erythroid Ter119-positive* ***(E)*** *and granulocyte Gr1-positive* ***(F)*** *progenitor cells are EGFP + in constitutive VE-Cadherin-Cre;mT/mG mice compared to Cre-negative;mT/mG-positive control mice. Abbreviations: VECreERT2, mTmG;VE-Cadherin-Cre-ERT2; VECre, mTmG;VE-Cadherin-Cre,.*

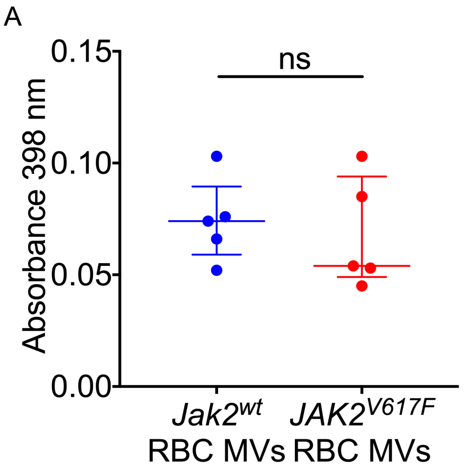

Figure *S2. Heme contained in erythrocytes microvesicles do not explain the NO pathway inhibition.*

Absorbance at 398nm (Heme) of microvesicles derived from Jak2$^{V617F\ Flex/WT}$ *;VE-Cadherin-cre mice (Jak2*^{V617F} *HC-EC, n=5, red) and control mice erythrocytes (Jak2*^{WT}*, n=5, blue). Data are expressed as median with interquartile range. Abbreviations: MVs, microvesicles; ns, not significant; RBC, red blood cells. Data were compared using the Mann-Whitney U-test. All tests were 2 sided.*

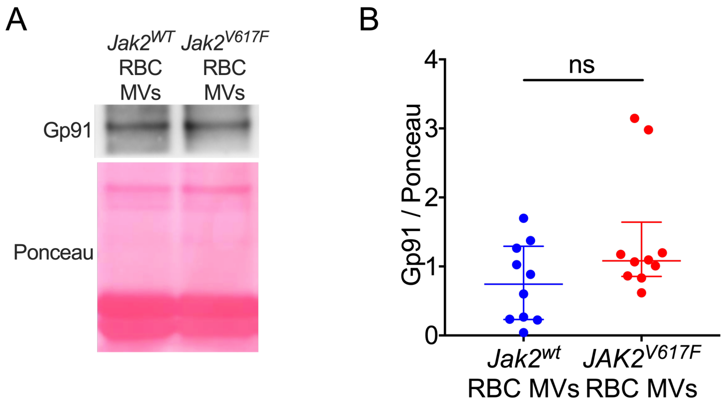

Figure *S3. Gp91 expression in erythrocyte-derived microvesicles*

Representative image ***(A)*** *with quantification* ***(B)*** *of Gp91 (NOX2) western blot performed on microvesicles derived from JAK2*^{WT} *(n=10, blue) and JAK2*^{V617F} *(n=10, red) erythrocytes. Quantitative data are expressed as median with interquartile range. Data were compared using the Mann-Whitney U-test. All tests were 2 sided. Abbreviations: ns, not significant.*

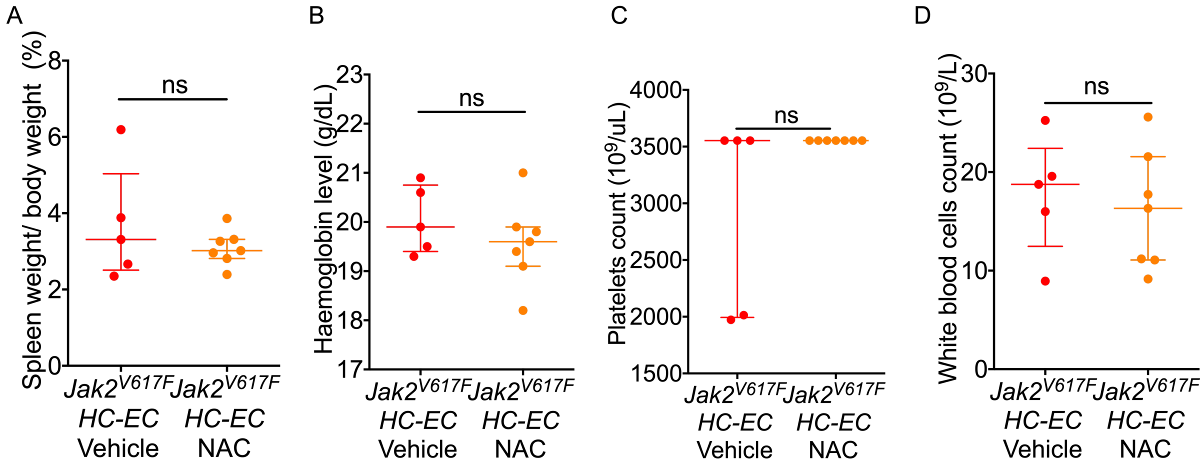

Figure *S4. Spleen weight and blood cell counts in N-acetyl-Cystein treated mice.*

Spleen weight/body weight ratio ***(A),*** *haemoglobin level* ***(B),*** *platelet count* ***(C)*** *and white blood cell count* ***(D)*** *from Jak2^{V617F} HC-EC mice treated with vehicle (n=5, red) and with NAC (n=7, orange). Data are expressed as median with interquartile range. Abbreviations: Jak2^{WT}, control mice; Jak2^{V617F} HC-EC, Jak2$^{V617F\ Flex/WT}$;VE-Cadherin-cre mice; NAC, N-Acetyl-cysteine; ns, not significant. Data were compared using the Mann-Whitney U-test. All tests were 2 sided.*

Table S1

Protein	Description	Ratio	p value	Peptides	Effect on oxidative stress	Ref	Deregulation potentially explaining the observed effect
sp|P07901|HS90A_MOUSE	Heat shock protein HSP 90-alpha	3.45	5.3e-38	128	Anti-Oxidant<br>Increased by ROS	*(61)*	No
sp|P11247|PERM_MOUSE	Myeloperoxidase	∞	-	38	Pro-Oxidant	*(22)*	**Yes**
sp|Q9JMH6|TRXR1_MOUSE	Thioredoxin reductase 1	2.66	1.4e-05	29	Anti-Oxidant	*(62)*	No
sp|Q64471|GSTT1_MOUSE	Glutathione S-transferase theta-1	1/2.25	3.6e-05	20	Anti-Oxidant	*(62)*	**Yes**
sp|P31725|S10A9_MOUSE	Protein S100-A9	4.28	4.1e-09	18	Anti-Oxidant<br>Increased by ROS	*(63)*	No
sp|P27005|S10A8_MOUSE	Protein S100-A8	3.95	5.5e-06	15			
sp|Q61133|GSTT2_MOUSE	Glutathione S-transferase theta-2	∞	-	10	Anti-Oxidant	*(62)*	No
sp|P02104|HBE_MOUSE	Hemoglobin subunit epsilon-Y2	1/ ∞	-	8	Pro-Oxidant	*(62)*	No
sp|O08807|PRDX4_MOUSE	Peroxiredoxin-4	∞	-	6	Anti-Oxidant<br>Increased by ROS	*(21)*	No
sp|P08226|APOE_MOUSE	Apolipoprotein E	∞	-	6	Anti-Oxidant	*(64)*	No
sp|Q9D975|SRXN1_MOUSE	Sulfiredoxin-1	∞	-	6	Anti-Oxidant<br>Increased by ROS	*(65)*	No
sp|P62897|CYC_MOUSE	Cytochrome c, somatic	∞	-	6	Anti-Oxidant	*(66)*	No
sp|P09671|SODM_MOUSE	Superoxide dismutase, mitochondrial	∞	-	5	Anti-Oxidant<br>Increased by ROS	*(66)*	No
sp|Q61093|CY24B_MOUSE	Cytochrome b-245 heavy chain	∞	-	5	Pro-Oxidant	*(62)*	**Yes**
sp|Q61462|CY24A_MOUSE	Cytochrome b-245 light chain	∞	-	5			
sp|Q69ZK0|PREX1_MOUSE	Phosphatidylinositol 3,4,5-trisphosphate-dependent Rac exchanger 1 protein	1/ ∞	-	4	Pro-Oxidant	*(62)*	No
sp|Q6ZPY7|KDM3B_MOUSE	Lysine-specific demethylase 3B	1/ ∞	-	4	Epigenetic regulation	*(67)*	No
sp|O35143|ATIF1_MOUSE	ATPase inhibitor, mitochondrial	1/ ∞	-	3	Pro-Oxidant	*(68)*	No

Table S1. List of proteins involved in oxidative stress and deregulated in microvesicles derived from JAK2^{V617F} as compared with JAK2^{WT} red blood cells, identified by using quantitative label-free mass spectrometry analysis

Cellular oxidant detoxification (GO 0098869) and ROS metabolic process (GO 0072593) proteins significantly deregulated ($p< 0.05$, and ratio JAK2^{V617F} / JAK2^{WT} > 2 or < 0.5, and with ≥ 3 peptides) or with peptides identified only in one sample type by using quantitative label-free mass spectrometry analysis performed from JAK2^{V617F} (6 samples) and JAK2^{WT} (4 samples) erythrocyte-derived microvesicles.
